# Gender and urban health: a Latin American structured tool for research and policy

**DOI:** 10.1590/0102-311XEN046124

**Published:** 2025-02-07

**Authors:** Lidia Maria de Oliveira Morais, Elis Borde, Paula Guevara, Roxana Valdebenito, Laura Baldovino-Chiquillo, Olga L. Sarmiento, Alejandra Vives Vergara, Amélia Augusta de Lima Friche, Waleska Teixeira Caiaffa

**Affiliations:** 1 Universidade Federal de Minas Gerais, Belo Horizonte, Brasil.; 2 Facultad de Medicina, Universidad de Los Andes, Bogotá, Colombia.; 3 Facultad de Medicina, Pontificia Universidad Católica de Chile, Santiago, Chile.

**Keywords:** Gender and Health, Gender-Inclusive Policies, Evidence-Informed Policies, Saúde e Gênero, Políticas Inclusivas de Gênero, Política Baseada em Evidências, Género y Salud, Políticas Inclusivas de Género, Política Informada por la Evidencia

## Abstract

Latin American cities have evolved via exclusionary historical processes, resulting in hasty and unplanned urbanization, insufficient infrastructure, and extreme levels of violence. These issues have well-documented health implications. In urban settings, gender may lead to unequal access to opportunities and services, however, its consideration into policies, interventions, and research remains insufficient, potentially exacerbating urban inequities. Drawing inspiration from feminist urbanism and urban health research, we propose a structured tool for Latin American cities to develop gender-sensitive urban policies, interventions, and urban health research. The study encompassed: (1) a narrative literature review of feminist urbanism frameworks and the Delphi method to select the most appropriate dimensions; (2) a thorough examination of data availability and indicators in three studies of urban transformation interventions in Brazil, Colombia, and Chile to evaluate data availability and local interest; and (3) an urban health dialogue with the relevant indicators. We identified three key dimensions: “proximity”, “autonomy”, and “representativeness”. Neighborhood was considered the most meaningful level for analyses. The indicators were organized into subdimensions, considering existing literature on their implications for gender and health. The proposed tool is comprehensive and adaptable, thus, it can be used in diverse Latin American urban contexts. It is a valuable resource for incorporating a gender-sensitive perspective into urban policymaking, interventions, and health-related research.

## Abstract (pt)

As cidades da América Latina evoluíram por meio de processos históricos excludentes, resultando em uma urbanização rápida e não planejada, com infraestrutura defasada e altos níveis de violência. Essas questões têm implicações bem documentadas para a saúde. Em ambientes urbanos, o gênero pode levar ao acesso desigual a oportunidades e serviços, no entanto, sua consideração em políticas, intervenções e pesquisas permanece insuficiente, potencialmente exacerbando desigualdades urbanas. Inspirando-nos no urbanismo feminista e na pesquisa em saúde urbana, propomos uma ferramenta estruturada para que as cidades latino-americanas desenvolvam políticas, intervenções e pesquisas em saúde urbana sensíveis ao gênero. O estudo abrangeu (1) uma revisão narrativa da literatura sobre os modelos do urbanismo feminista e um método Delphi para selecionar as dimensões mais adequadas; (2) um exame minucioso da disponibilidade de dados e indicadores em três estudos de intervenções de transformação urbana no Brasil, Colômbia e Chile para avaliar a disponibilidade de dados e o interesse local; e (3) um diálogo sobre saúde urbana com indicadores relevantes. Identificamos três dimensões principais: “proximidade”, “autonomia” e “representatividade”. O bairro foi considerado o nível mais significativo para as análises. Os indicadores foram organizados em subdimensões, considerando a literatura existente sobre suas implicações para gênero e saúde. A ferramenta proposta é abrangente e adaptável, atendendo aos diversos contextos urbanos da América Latina. Além disso, trata-se de um recurso valioso para incorporar uma perspectiva sensível ao gênero na formulação de políticas urbanas, intervenções e pesquisas relacionadas à saúde.

## Abstract (es)

Las ciudades de América Latina han evolucionado mediante procesos históricos excluyentes, que resultaron en una urbanización rápida y no planificada, con infraestructura obsoleta y elevados niveles de violencia. Estas cuestiones tienen implicaciones para la salud bien documentadas. En entornos urbanos, el género puede conducir a un acceso desigual a oportunidades y servicios, pero su consideración en las políticas, intervenciones e investigaciones sigue siendo insuficiente, lo que podría exacerbar las desigualdades urbanas. Inspirándonos en el urbanismo feminista y en la investigación en salud urbana, proponemos una herramienta estructurada para que las ciudades latinoamericanas desarrollen políticas, intervenciones e investigaciones en salud urbana sensibles al género. El estudio abarcó (1) una revisión narrativa de la literatura sobre los marcos del urbanismo feminista y un método Delphi para seleccionar las dimensiones más adecuadas; (2) un examen exhaustivo de la disponibilidad de datos e indicadores en tres estudios de intervenciones de transformación urbana en Brasil, Colombia y Chile para evaluar la disponibilidad de datos y el interés local; y (3) un diálogo sobre salud urbana con indicadores relevantes. Identificamos tres dimensiones principales: “proximidad”, “autonomía” y “representatividad”. El barrio fue considerado el nivel más significativo para los análisis. Los indicadores se organizaron en subdimensiones, considerando la literatura existente sobre sus implicaciones para el género y la salud. La herramienta propuesta es integral y adaptable, y atiende a los diversos contextos urbanos de América Latina. Además, se trata de un recurso valioso para incorporar una perspectiva sensible al género en la formulación de políticas urbanas, intervenciones e investigaciones relacionadas con la salud.

## Background

Latin American cities have evolved via exclusionary historical processes, resulting in unplanned and accelerated urbanization, environmental degradation, lacking infrastructure, and widespread ^1^ ^,^ ^2^. There is increasing recognition of the need to harness urban change to foster equity and develop inclusive planning strategies, enhancing community engagement, prioritizing equitable resource distribution, and promoting health and well-being ^3^.

Herein, gender inequities are also increasingly recognized as determinants of health and unequal access to opportunities and services. Previous studies have shown how the gendered individual experience of urban built environments affects people’s sense of security, mental health, workload, care work, mobility, time allocation, and physical activity ^4^ ^,^ ^5^ ^,^ ^6^ ^,^ ^7^ ^,^ ^8^ ^,^ ^9^. While we can define sex as a category attributed at birth and defined by the individual’s physiological, genetic, and phenotypic characteristics ^10^, gender is relational. It interacts with historically determined cultural roles, socioeconomic profiles, race, religion, and other individual and social factors ^11^ ^,^ ^12^, which implies social expectations and identity hierarchies. Gender isa socio-cultural construct ^13^ ^,^ ^14^. This debate is far more complex since sex is also a culturally-shaped definition ^15^, and gender challenges binary ideals of women and men as a constantly evolving individual performance, as argued by Butler ^16^. What is important for us here is they are both essential determinants of health and affect urban living ^15^ ^,^ ^16^.

In this direction, Feminist Urbanism scholarship provoked important discussions on how cities are projected and transformed without due consideration of any gender identities besides the “standard” man and their typically attributed social roles ^17^ ^,^ ^18^. They have also provided evidence on how the urban environment materializes and reproduces gender inequities. According to relevant literature ^19^ ^,^ ^20^ ^,^ ^21^ ^,^ ^22^ ^,^ ^23^ ^,^ ^24^ ^,^ ^25^ ^,^ ^26^ ^,^ ^27^, the built environment may facilitate access to resources and amenities relevant to the daily routines associated with stereotypical male roles, such as working outside the household, using more motorized transport, and practicing more sports, but not those attributed to women, as unpaid care work, household chores, nonmotorized transport, and informal work ^28^ ^,^ ^29^ ^,^ ^30^ ^,^ ^31^. Symbolically, street names and public monuments are mainly referring to men and man-related accomplishments, as an expression of gender inequities and gender-biased urbanization ^32^.

Feminist Urbanism scholars have developed frameworks and practical tools to evaluate urban settings based on a gender-sensitive perspective. Generally, they argue that flexible spaces that encompass diverse users’ needs tend to favor the coexistence, interaction, and circulation of people and potentially promote gender equity ^8^ ^,^ ^19^ ^,^ ^22^ ^,^ ^33^ ^,^ ^34^ ^,^ ^35^. Most of these frameworks were developed based on Global North experiences, not accounting for cities specificities and the lived experiences of urban dwellers in the Global South. They have also predominantly addressed the effects of unequally gender-planned urban environments on mobility ^36^ ^,^ ^37^ ^,^ ^38^ ^,^ ^39^, sense of belonging ^40^ ^,^ ^41^ ^,^ ^42^, and other expressions of urban life. However, only a few directly acknowledge possible health effects ^23^, not yet covered by Urban Health literature.

Urban Health is the discipline within Public Health that studies the risk factors inherent to living in urban environments, the built and social environments, and their effects on health ^43^. Although Urban Health frameworks ^13^ ^,^ ^44^ have incorporated gender and there is growing recognition of the ways urban spaces produce different opportunities and constraints for men, women, and other gender identities ^45^ ^,^ ^46^ ^,^ ^47^ ^,^ ^48^ ^,^ ^49^, Urban Health literature has largely failed to adequately consider the gendered impacts of urban living on health ^14^ ^,^ ^50^ ^,^ ^51^ ^,^ ^52^. Most studies in the field do not sufficiently explore, for example, the gendered dynamics of urban violence ^53^, the health benefits of green spaces ^54^, and the effects of neighborhood “disorder” ^55^. As gender-sensitive indicators are often missing, sex data is used as a proxy, and both terms appear interchangeably ^13^ ^,^ ^44^. Health data is often not even disaggregated by sex, implying that gender discussions are absent or only marginally present in Urban Health study designs, methodologies, and results interpretation.

In a dialogue between Urban Health and Feminist Urbanism, we propose a data-oriented structured tool to help guide researchers and policymakers into gender-sensitive urban health research and policies in Latin America.

## Methods

To build the tool, a narrative literature review was conducted to identify and characterize relevant Feminist Urbanism frameworks. Secondly, a detailed guiding framework was selected and the Delphi method was used ^56^ with urban health researchers to identify which of the framework dimensions and indicators best suited Urban Health research purposes. Finally, the datasets of three quasi-experimental mixed-methods Urban Health studies on the health impacts of urban transformation interventions in Brazil, Colombia, and Chile ^57^ ^,^ ^58^ ^,^ ^59^ were examined to identify indicators and data sources that could possibly inform gender-sensitive analyses. Then, the *Gender-sensitive Latin American Urban Health Research and Policy Tool*, in short, *Gender Latin American Tool* organized by dimensions and subdimensions was developed.

### Literature review and framework selection

A narrative literature review is a suitable method for synthesizing and interpreting existing literature, especially in emerging or multidisciplinary research areas where the evidence is varied and still developing ^60^. Even though it is not a comprehensive review, it is rigorous and flexible enough to allow the dialogue between disciplines and may be shaped to fit research specificities ^61^ ^,^ ^62^ ^,^ ^63^ ^,^ ^64^.

Given the lack of gender approaches in Urban Health and the availability of some Feminist Urbanism tools and frameworks to objectively evaluate the urban environment with gender-sensitive lens, a broad narrative literature search was conducted to identify tools and frameworks that would dialogue with Urban Health scholars and the available data. This narrative literature review was performed in 2022, firstly using academic literature search engines and secondly, hand-searching each of the identified publications reference lists. Finally, urban health and feminist urbanism specialists were consulted to validate the identified tools. The full publications list is available in the Supplementary Material 1 (https://cadernos.ensp.fiocruz.br/static//arquivo/supl-e00046124_1869.pdf).

Both academic and gray literature (reports, handbooks, dissertations, websites), in English, Spanish, and Portuguese, with no established period were included and one of the 35 identified tools was selected due to its detailed structure and indicators. As a second step, the Delphi method ^56^ was used to prioritize select the most relevant indicators and dimensions to compose the Latin American tool. Details on the narrative review and Delphi method can also be found in the Supplementary Material 1 (https://cadernos.ensp.fiocruz.br/static//arquivo/supl-e00046124_1869.pdf).

### Data availability and gender-sensitiveness of Latin American studies indicators

As a third step in the development of the *Gender Latin American Tool*, the datasets of three quasi-experimental mixed-methods Latin American urban health studies were examined to identify possible indicators and data sources that could inform gender-sensitive analyses. The studies conducted as part of the *Salud Urbana en America Latina* (SALURBAL) project ^65^ specifically addressed the effects of urban transformation interventions in Brazil, Chile, and Colombia, namely: *PAC Vila Viva Program* (PVV) in Belo Horizonte State and the BH-Viva ^58^; *Programa de Regeneración de Conjuntos Habitacionales* in Viña del Mar and Puente Alto and the *Regeneración Urbana, Calidad de Vida y Salud* (RUCAS) ^59^; and *TransMiCable* in Bogotá and the *Transformaciones Urbanas y Salud: El Caso de TransMiCable* (TrUST) ^57^. Interventions included housing projects and changes to the neighborhood social and built environment, mainly linked to public services and the implementation of facilities, pavement and road widening, leisure, as well as the installation of sports facilities, transport and mobility improvements, and community-level social interventions. The studies used quantitative and qualitative methods and pre- and post-intervention measures ^10^ ^,^ ^57^ ^,^ ^58^ ^,^ ^59^ ^,^ ^66^ ^,^ ^67^ ^,^ ^68^ ^,^ ^69^.

The studies structure and available indicators were analyzed, disregarding their outcomes. Details on the SALURBAL studies that informed the tool development including location, affected populations, and methods are available in the Supplementary Material 2 (https://cadernos.ensp.fiocruz.br/static//arquivo/supl-e00046124_1869.pdf). No study had specific gender-sensitive indicators. Therefore, this review aimed to identify studies that, based on Latina American Urban Health and Feminist Urbanism literature, could be considered sensitive to sex/gender markers.

### Gender-sensitiveness and urban health: grounding the selected indicators

Despite the differences in research design, the three intervention transformation studies presented relatable indicators ^70^ ^,^ ^71^ ^,^ ^72^, making it possible to organize them around dimensions, and subdimensions proposed in the selected framework. Indicators that were considered as gender-sensitive and that featured in at least two of the three studies were included in the *Gender Latin American Tool* synthesis, grounding the tool into currently available indicators in Latin American urban contexts.

The SALURBAL study protocol was approved by the Drexel University Institutional Review Board with ID #1612005035. In Brazil its register is CAAE 70209917.0.0000.5149 and the BH-Viva study CAAE: 11548913.3.0000.5149. RUCAS study is registered in Chile as Acta n. 806-2017; Acta n. 977-2019; Acta n. 994-2019) and TrUST as ID 170727004 in Colombia in their respective review boards. Informed consent was obtained from all subjects involved in the studies.

## Results and discussion

### Literature review and framework selection

Most publications we found focused on a specific theme, such as violence, transportation, or housing. Only a few had a broader perspective and encompassed multiple urban-life domains. Among these, we selected one that presented a framework with detailed dimensions and indicators, which would resonate with the ones commonly used in urban health research ^26^ ^,^ ^73^ ^,^ ^74^.

To guide our *Gender Latin American Tool* development we selected the framework *diagnóstico urbano con perspectiva de género* (DUG - urban diagnosis with a gender perspective) ^26^ ^,^ ^73^ ^,^ ^74^. We used the Delphi method to guide the neighborhood selection as the most relevant spatial level for built environment influence, and the selection of three out of the five original characteristics - proximity, autonomy, and representativeness - to compose our Latin American tool. These characteristics, applied to the neighborhood level, are named hereinafter dimensions, unfold into subdimensions and suggested indicators. Supplementary Material 1 (https://cadernos.ensp.fiocruz.br/static//arquivo/supl-e00046124_1869.pdf) shows detailed results of the Delphi method.

The three dimensions were originally defined as follows: (1) proximity refers to the quality of the space that captures whether people can meet and relate to each other, access facilities and services, and the easiness to use public transport and shops. It considers that all daily activities should be reachable by walking and public transportation, considering the reasonable distance and time allocation for all types of people, guaranteeing different uses of space ^26^ ^,^ ^74^; (2) autonomy captures how places provide people with sense and perception of safety, without restrictions to their use by anyone, regardless of one’s physical characteristics ^26^ ^,^ ^74^; and (3) representativeness assesses visibility, in both material and symbolic ways, highlighting the community’s memory and social and cultural heritage and fostering community participation in urban policy decisions ^26^ ^,^ ^74^.

### Data availability, gender-sensitiveness, and urban health: grounding the selected indicators

In the subsequent sections, we provide definitions for our dimensions and subdimensions, based on a thorough examination of data availability and indicators identified as possibly gender-sensitive in the three guiding urban transformation studies. They are presented in dialogue with relevant literature on Urban Health, presenting their potential for gender sensitiveness and health promotion in research and policymaking. Box 1 shows the selected indicators for each dimension and study.

**Box 1:** Proximity indicators per subdimension and data source of three included studies.

SUBDIMENSION/STUDY	HOUSEHOLD SURVEY	QUALITATIVE COMPONENT	BUILT ENVIRONMENT SYSTEMATIC OBSERVATION
	Indicator	Theme	
Access and use of services and facilities			
BH-Viva	Frequency of use of primary health care facilities	Access to services and facilities	No data available
RUCAS	Frequency and duration of use of the neighborhood park	No data available	Number of park visitors per park areas, available recreational areas, male/female visitors
	Frequency and duration of use of the neighborhood sports court		
TrUST	Walking time to access the police stations	Access to services and facilities	Park area considered accessible, usable, empty, equipped
	Access to banks in less than 20 minutes on foot		
	Walking time to access facilities, pharmacies, and supermarkets		
	Walking time to access leisure and sport facilities	Perception of the parks	
	Walking time to access parks and green areas		
Perception of public transport			
BH-Viva	Perception of public transport service	Transport availability	Bus stops and quality of related street furniture
RUCAS	No data available	Transport availability	No data available
TrUST	Perception of public transport service	TransMiCable perception	No data available
Perception of sidewalk quality			
BH-Viva	Perception of presence and quality of sidewalks	No data available	Presence of sidewalks
TrUST	Presence of sidewalks close to public transportation facilities	Quality of sidewalks	No data available
Street maintenance			
BH-Viva	Street maintenance (presence of vacant lots, garbage, rubble, with tall weeds)	No data available	Presence of weed, wholes, garbage, among others, in the sidewalks
TrUST	Perception of pavement quality	Garbage management	No data available

BH-Viva: the study of *Vila Viva Program* (PVV); RUCAS: *Regeneración Urbana, Calidad de Vida y Salud*; TrUST: *Transformaciones Urbanas y Salud: El Caso de TransMiCable*.

#### Proximity

Proximity is most directly related to the characteristics of the built environment, considering aspects of distance, location, and accessibility. It is determined by how different social groups are placed within the city and neighborhood, how these are spatially organized, and what effort is necessary for different dwellers to carry out daily routine activities (formal work and care work, for example) ^26^ ^,^ ^29^ ^,^ ^38^ ^,^ ^74^.

The three evaluation studies contained information on the frequency of use and distance (walking time) to healthcare, schools, childcare, commercial facilities, banks, police stations, parks, and sports courts, for example (Box 1).

Looking at available indicators through a gender lens can reveal disparities in accessing certain amenities that are influenced by gender-based mobility patterns and societal expectations of gender roles. For example, pieces of literature indicate that women and girls tend to use parks and green spaces less frequently due to their daily routines, and concerns about safety and potential violence in those areas. This highlights the need to address gender-based barriers to equal access ^75^ ^,^ ^76^.

Evidence suggests transportation modes are also gender sensitive. Studies have shown that differences in transport accessibility can worsen gender and socioeconomic inequalities, particularly for women who often have less access to individual motorized vehicles and are disproportionately reliant on public transportation ^6^. Issues such as low-quality public transportation and sexual harassment can act as barriers for women seeking access to opportunities, services, and facilities ^5^. The examined urban intervention studies include transportation-related indicators and could potentially explore gendered effects of urban transformation interventions focusing on traffic, possibly revealing differential effects of interventions focusing on public, individual motorized and active modes of transportation, respectively.

Studies indicate that women in Latin American urban environments tend to walk more than men ^6^ ^,^ ^36^ ^,^ ^37^, so we should observe walking for its gender sensitiveness. Box 2 lists the indicators related to this topic, namely: sidewalks presence/absence of sidewalks and their maintenance quality, marked by the presence of garbage and weeds presence, and lack of or bad-quality pavement, along with street maintenance. Differences in these indicators may be related to gendered walking routines but also to differential needs considering, for example, strollers, wheelchairs, and other tools used in care work are pushed mainly by women ^39^ ^,^ ^77^.

**Box 2:** Autonomy indicators per sub dimension and data source of three included studies.

SUBDIMENSION/STUDY	HOUSEHOLD SURVEY	QUALITATIVE COMPONENT	BUILT ENVIRONMENT SYSTEMATIC OBSERVATION
	INDICATOR	THEME	
Perception of safety while walking in the public spaces			
BH-Viva	Perception of safety while walking during the day	Perception of safety	Presence of police officers and police stations
	Perception of safety while walking during the night		
RUCAS	Perception of safety while walking during the day	Perception of unsafety	No data available
	Perception of safety while walking during the night		
Perception of unsafety			
BH-Viva	Perception of crime/violence in the neighborhood	Perception of violence/safety	No data available
RUCAS	Perception of drug use/trafficking in the neighborhood	Perception of unsafety	Presence of drug trafficking in parks
	Perception of frequency of robberies and assaults in the neighborhood		
	Perception of frequency of verbal harassment in the neighborhood		
TrUST	Perception of unsafety in the neighborhood	Perception of unsafety	No data available
Unsafety during or around transportation			
RUCAS	Perception of motorized vehicles in high speed	No data available	No data available
	Perception of unsafety while waiting for public transport		
TrUST	Perception of unsafety due to vehicle traffic on the walking route to public transport	No data available	No data available
	Robberies in the walking route to public transport		
Public spaces lighting			
BH-Viva	Street lighting	No data available	Presence of streetlights
RUCAS	Street lighting	Quality of street lighting	No data available
TrUST	Satisfaction with street lighting	No data available	No data available
	Presence of street lighting		
Perception of the police			
BH-Viva	Perception of police presence and quality	Police perception	Presence of police officers, vehicles, and police stations
TrUST	Trust in the police	Police perception	No data available
Perception of safety for children			
BH-Viva	Friendly neighborhood for children	Children’s exposure to unsafe situations	Presence of children playing or engaged in physical activities on the street
	Children playing outside the house		
RUCAS	Safe neighborhood for children to play	No data available	No data available

BH-Viva: the study of *Vila Viva Program* (PVV); RUCAS: *Regeneración Urbana, Calidad de Vida y Salud*; TrUST: *Transformaciones Urbanas y Salud: El Caso de TransMiCable*.

By exploring the proximity dimension, one observes that adopting a gender perspective is necessary to move beyond a simplistic and merely numeric understanding of proximity to services and facilities in public policies and urban planning. This is especially the case of how proximity and distance are relational and profoundly shaped by the perception of the built environment ^26^ ^,^ ^74^ ^,^ ^78^.

#### Autonomy

The autonomy dimension reflects the social environment by synthesizing the ability to move around without feeling threatened or restricted for safety reasons ^6^ ^,^ ^79^. This dimension is a relevant marker of gender equity, especially in Latin America, where gender-related violence reaches alarming levels ^1^ ^,^ ^80^ ^,^ ^81^ ^,^ ^82^. Thus, perceptions of safety, violence, or crime in the neighborhood depend on people’s gender identity ^83^ ^,^ ^84^ ^,^ ^85^ ^,^ ^86^ ^,^ ^87^ and unequally motivate or inhibit autonomy in daily routines. In the examined case studies, this dimension is potentially informed by indicators such as security or violence/crime perception, street lighting, policing or drug trafficking presence, and heavy motorized vehicles traffic, which can influence how public spaces can be welcoming or threatening ^88^ ^,^ ^89^ ^,^ ^90^ ^,^ ^91^ (Box 2).

According to the literature, fear of violence limits healthy behaviors such as walking, spending time outside at parks and leisure facilities, or letting children play in the neighboring areas ^88^ ^,^ ^89^ ^,^ ^91^. In the examined studies, gender dimensions of inadequate street lightning and the presence of motorized vehicles in heavy traffic could be considered as sensitive markers of gender-(in)equitable environments, as both aspects potentially limit autonomy and also the possibilities of healthy behavior of both women and children.

Transformation interventions in Latin American urban-built environments have encompassed, for example, the widening of streets and alleys, aiming at enhancing visibility, reducing hiding spots for potential aggressors, and facilitating law enforcement. It is essential to recognize that the outcomes of these transformations have gendered implications as women, men, and people with non-binary gender identity experience urban violence differently. As displayed in Box 2, the examined studied included indicators on safety perception, which can be a useful starting point to explore gender differences in the perceptions of fear of crime, and how this can impact overall health ^92^ ^,^ ^93^.

We must highlight that violence and crime are sensitive matters to discuss in research interviews as the fear of violence from close partners and neighbors, shame and stigma may create bias and underreporting.

Overall, we can infer built environments that favor visibility, connection between neighbors, trust, and familiarity, may encourage a general perception of safety and promote gender equity and healthier environments.

#### Representativeness

Representativeness plays a multifaceted role in evaluating gender equity, encompassing tangible and symbolic aspects that contribute to an individual’s satisfaction with their neighborhood and sense of belonging. Given the nuanced nature of urban interventions, there is a compelling need for a gender-sensitive approach. These interventions can positively affect the sense of belonging and satisfaction by enhancing neighborhood aesthetics and mitigating previous feelings of stigma associated with residing in the area ^94^. Box 3 shows several indicators that evaluate this dimension found in the included studies.

**Box 3:** Representativeness indicators per sub-dimension and data source of three included studies.

SUBDIMENSION/STUDY	HOUSEHOLD SURVEY	QUALITATIVE COMPONENT
INDICATOR	THEME
Neighborhood satisfaction		
BH-Viva	Intention to continue living in the neighborhood	Eviction/reallocation
RUCAS	Overall satisfaction with the neighborhood	Stigmatization
TrUST	Satisfaction of living in the neighborhood	Satisfaction
Sense of belonging		
BH-Viva	Feeling of belonging	Social network
RUCAS	Feeling of belonging	Feeling of unity and trust
TrUST	No data available	Community strength

BH-Viva: the study of *Vila Viva Program* (PVV); RUCAS: *Regeneración Urbana, Calidad de Vida y Salud*; TrUST: *Transformaciones Urbanas y Salud: El Caso de TransMiCable*.

As outlined by López-Contreras et al. ^42^, neighborhood satisfaction, within the context of the built environment, involves evaluating factors such as the overall neighborhood attractiveness and cleanliness and evaluating the quality of spaces where social interactions occur. Notably, neighborhood satisfaction can significantly impact individuals’ lives, particularly those predominantly responsible for daily caregiving tasks which, in Latin America, are often women. Conversely, a sense of belonging is characterized by individuals experiencing a solid connection and trust in their neighbors and a deep attachment to the place and its residents ^42^. This sense of belonging is strongly associated with feeling welcomed, identifying with peers, and connecting with the surrounding environment ^40^ ^,^ ^41^ ^,^ ^42^.

Urban living frequently affects social bonds and can lead to sense of isolation and individualism. This is particularly pertinent in Latin America for Indigenous, poor, and Black women, who have historically faced segregation resulting from structural racism, classism, and sexism, hindering their access to public services and facilities, making communities, neighbors, and families their primary support network ^12^ ^,^ ^95^. A sense of belonging is often linked to a historical bond with the neighborhood and social network due to extended periods of residence.

Urban transformation interventions can also exacerbate gender inequities, as elucidated by Nakhal ^9^ (p. 16) “*Standardization specifically leads to a lack of security and safety on one level and an environment that reinforces existing power dynamics, thereby perpetuating the patriarchal character of space*”. Other experiences include “*feelings of powerlessness in the regeneration process, increased levels of stress and uncertainty, and genuine concerns about being unable to return to the estate after regeneration*” ^96^ (p. 618). Also, individuals who have been evicted and relocated to make way for urban interventions may be more susceptible to experiencing detrimental effects on their satisfaction and sense of belonging. Such changes can lead to losing familiar references and symbolic identification with the territory.

Representativeness is closely intertwined with both proximity and autonomy. If these two dimensions receive favorable ratings, representativeness will likely follow suit, reflecting overall satisfaction with the neighborhood as a health-enhancing, or at least non-degrading, environment for individuals of different genders and with diverse needs.

### A gender sensitive Latin American urban health research and policy tool

As argued above, we propose a method to encompass Feminist Urbanism and Urban Health literature, looking into the availability of built environment indicators in Latin American contexts to propose a hands-in tool to support research and policymaking. This tool can be synthesized in a simple box featuring dimensions, subdimensions, and suggested priority indicators that can be incorporated in Urban Health study designs and in more generic planning processes and evaluation studies of urban interventions. Note that the tool does not seek to be exhaustive, and other indicators can be added to adapt to local context data availability and specificities. Box 4 details the structured tool. Along with the tool, we suggest that any urban health research and policymaking in Latin America include women and diverse gender identities, as well as concerns with race, age, and ability diversity, in the design, planning, and execution of urban projects.

**Box 4:** Gender-sensitive Latin American Urban Health research and policy tool: Synthetic dimensions and subdimensions definitions and indicators.

DIMENSION	DEFINITION	SUBDIMENSIONS	INDICATORS
Proximity	Quality of the space that captures if people can meet and relate to each other, access facilities and services, and the easiness of use of public transport and shops. Note that all daily activities should be connected in distance and time, especially by walking and public transportation, for all types of people, guaranteeing the different uses of space.	Access and use of services and facilities	Frequency of use, time walking to the facility
		Perception of public transport	Quality or frequency of available public transport
		Perceptions of sidewalk quality	Presence and quality of maintenance
		Street maintenance	Presence of garbage, weeds, pavement
Autonomy	Quality of the space that captures how places provide people with feelings and perception of safety, without restrictions to their use by anyone, regardless of one’s physical characteristics.	Perception of safety while walking in public spaces	Safety perception during the day/nigh
		Perception of unsafety in the neighborhood	Perception of the frequency of various crime signs
		Unsafety during or around transportation	Fear of crime/violence or heavy motorized traffic
		Children’s safety perception	Play outside, fear of crime/violence
		Public spaces lighting	Presence, satisfaction
		Perception of the police	Presence, trust, and relationship with the community
Representativeness	Quality of the space that captures visibility, in both material and symbolic ways, highlighting the community’s memory and social and cultural heritage, and fostering community participation in urban policy decisions.	Satisfaction with neighborhood	Overall satisfaction, intention to continue living in the neighborhood
		Sense of belonging	Social network, sense of belonging

### Limitations

Our limitations are related to the scarcity of Latin American approaches in the relevant literature and on the fact that data is usually limited to sex and not gender - not to mention that transgender or nonbinary gender identities are not even included in most databases.

Most feminist Urbanism studies we found were based on Global North frameworks, posing challenges for the adaptation to the Global South and, more specifically, Latin American cities. There is, however, growing interest in the region ^35^ ^,^ ^36^ ^,^ ^37^ ^,^ ^89^ ^,^ ^90^ ^,^ ^97^ ^,^ ^98^ ^,^ ^99^ ^,^ ^100^, and this work contributes to that.

We have argued that it is possible to integrate gender-sensitive analyses in studies and policies that were not originally designed to observe gender inequities. Nevertheless, we stress that future studies must include gender and gendered roles rather than sex data, which would enable more in-depth analyses of the intertwinement between gender and other dimensions of inequities. Also, although this study and the tool propose an important gender discussion, we must acknowledge that the data - and therefore our discussion - do not sufficiently include considerations on nonbinary gender identities.

Latin American cities are largely different regarding sizes, demographic conditions, nuances in ethnic and racial tensions, and specific political contexts that can pose methodological challenges. Nevertheless, we found several similarities across Latin American cities and urban interventions, which corresponds to a shared history of colonization, exclusionary urbanization processes, predominance of neoliberal urban development models, and a patriarchal structure that tends to build cities made by and for men ^17^.

## Final remarks

Gender represents a fundamental determinant of urban health and deserves a more comprehensive and directed approach. We have paved the way to interdisciplinary learning, bringing Feminist Urbanism literature to Urban Health research and policy applications. We proposed a structured tool for gender-sensitive research, policies and transformations in Latin American urban contexts. Proximity, autonomy, and representativeness dimensions are the benchmarks for the analysis, populated with subdimensions and respective exemplary indicators - to be adapted to local context specificities. Thus, we hope to offer a tool to more gender-equitable and healthier urban research and policymaking.
