# Supplementary material for: Gender and urban health: a Latin American structured tool for research and policy
Source: Cad Saude Publica. 2025 Feb 7;40(12):e00046124. doi: 10.1590/0102-311XEN046124 (PMC11805525; doi:10.1590/0102-311XEN046124)
Supplement: Supplementary file 1 [file 1678-4464-csp-40-12-EN046124-s.pdf]

Supplementary Material 1

Literature review and tool selection method

The narrative literature review

A narrative literature review is a suitable method "for synthesizing and interpreting existing literature, especially in emerging or multidisciplinary research areas where the evidence is varied and still developing"[1] and is a valuable resource for gaining a broad understanding of a topic and appreciating its complexity and nuances. Unlike systematic reviews that involve a detailed and explicit protocol for literature search, selection, data extraction, and analysis, in narrative reviews the search is more flexible and although the search may not be exhaustive, it is perfectly suited to provide a comprehensive overview of a topic and explore its conceptual and theoretical dimensions[2–5].

Given the lack of gender approaches in urban health and the limited availability of feminist urbanism tools and frameworks to objectively assess the urban environment with a gender-sensitive lens, we, therefore, opted to perform a narrative literature review, which seemed more suitable to achieve the general study objectives.

The narrative literature review was performed in 2022 following five steps:

First, to identify adequate search terms, we consulted the MeSH (Medical Subject Headings) system. The only relevant descriptors we identified were 'Feminism' and 'Gender' which we deemed too unspecific. We, therefore, expanded the search and consulted the DeCS which listed 'Feminism' and 'Gender' but the latter always as a combined term such as in 'gender identity' or 'gender role'. The same applied for 'Urban' which was also only listed as a combined term, such as in 'urban renewal', 'urban population', and 'urban area'.

Second, as the indexed descriptors were not suiting the objectives of the literature search, we opted to broaden the search terms and performed a broad search in English, Portuguese, and Spanish in the research databases PubMed and Google Scholar using the terms 'Feminist urbanism' AND 'Tool' OR 'Framework', which resulted in rather generic results. To complement this search and guarantee a broad identification of relevant publications and more specific feminist urbanism tools and frameworks, as a fourth step, we expanded the search, substituting the original terms for more generic ones, looking for 'Gender' OR 'Feminist', AND 'Urban', AND 'Evaluation' OR 'Assessment' OR 'Tool' OR 'Framework', following with title and abstract-screening for any feminist urbanism framework or tool that has not been identified in our previous steps. We hand-

searched the reference lists of all identified publications for any other possibly interesting publication.

And at last, the universe of publications was reviewed by both urban health and feminist urbanism specialists, which have also indicated literature that was included in the narrative review by that time.

Scientific and gray literature (reports, handbooks, dissertations, websites) were included without publication date restrictions.

Among 41 identified publications we selected 35, which had (1) objective assessment of urban environment characteristics and (2) gender considerations beyond sex variables. They are presented in Table 1.

All selected publications were title and abstract screened by three of the authors evaluating the inclusion criteria: (1) objective assessment of urban environment characteristics and (2) gender considerations beyond sex variables. If considered eligible, full texts were analyzed, and organized by the objective measures or criteria they used to evaluate the urban environment from a feminist urbanism perspective. At least two of the authors read each document independently.

Some of the publications focused on violence[6–9], others concentrated on specific health outcomes such as mental health[10], transportation[11–20], or housing[21,22]; some offered structured conceptual frameworks without a detailed description of the indicators to be evaluated[23–37], two were reviews on gender studies in urban spaces [10,15,36] and some focused explicitly on the concept of care[12,17,18,21,37,38]. Among all those, some were Latin American focused on specific themes[6,9,14,16–19,32,35]. We also found information on gender-sensitive urban evaluation experiences implemented in LA cities, but the methods were unavailable[20].

In a team meeting all publications that presented objective tools for the assessment of the build environment were discussed collectively, leading to the selection of one tool, described in three of the publications reviewed, that would better suit our study proposal: the Diagnóstico Urbano con Perspectiva de Género (DUG) or "Urban diagnosis With a Gender Perspective" (free translation from the original), a detailed and structured evaluation tool for a gender-sensitive assessment of the urban environment[39–41].

The DUG instrument is composed of thirteen indicators, organized around three spaces: Neighborhood and daily network; Relationship spaces; and Daily facilities (free translation from the original three espacios: Barrio y Red cotidiana, Espacio de relación, and Equipamiento cotidiano); and five characteristics: Proximity, Diversity, Autonomy, Vitality and Representativeness (free translation from the original five características: Proximidad, Diversidad, Autonomnia, Vitalidad, and Representatividad)[39–41].

For its detailing in the evaluation criteria and presenting indicators likely to be sensitive to LA build environment characteristics, the DUG was considered the most adequate to inform the development of this study.

The DELPHI procedure

The DUG instrument was then worked upon within a DELPHI procedure[42] for simplification and adaptation to Latin American urban contexts and available indicators that would possibly compose a gender-sensitive tool for research and policymaking. The DELPHI procedure was organized in two-rounds and with the participation of nine experts in interest areas (urban health, urban violence, spatial analysis, gender, qualitative analysis of health behaviors, urban interventions and regeneration, health and employment, and non-communicable diseases). All of them identified sex/gender as women/feminine, and were between 22 and 65 years old, equally distributed in junior and senior levels of expertise.

In the DELPHI 1st round, experts answered an online self-applied questionnaire with two main activities. Considering the broad question: "How do urban transformation interventions affect gender inequities, and what are their implications for health and health inequities in urban contexts in Latin America?". First, participants were asked to evaluate the level of daily influence of different scales of the built environment on both (i) health and (ii) gender inequities of people living in Latin American cities. In this step, the scales 'Barrio' and 'Vecindario' (possibly translated as neighborhood) were pointed as the most directly influencing health and gender inequities, followed by 'Vivienda' (housing), 'Territorio' (territory), and 'Ciudad' (city).

Then, participants were asked to classify the 13 indicators proposed by Ciocoletto[39–41] on a one (not or almost not important) to five (essential) Likert scale. In this step, 7/13 indicators were selected based on their classification as essential or very important by all or most of the participating experts. The selected indicators were part of the original urban qualities of Proximity, Autonomy, and Representativeness.

The final set of indicators was presented to the experts in DELPHI's 2nd round when, within a group discussion, the indicators selected were debated and agreed upon as being relevant to inform a Latin American gender-sensitive tool for evaluating the urban environments. Also, the indicators were evaluated facing the availability of health indicators from local Latin American urban intervention studies, matter of expertise of most participants involved, in a collective brainstorming. In the end, a final set of indicators was validated for being encompassing enough, the procedure having reached a consensus level, and thus achieving the DELPHI procedure objective.

As a result of the DELPHI procedure, the neighborhood level was considered the most relevant spatial level of influence, and three out of the five original characteristics, namely Proximity, Autonomy, and Representativeness, were considered priority. For our LA approach, these

characteristics, applied to the neighborhood level, are hereinafter named *Dimensions*. Characteristics of both Diversity and Vitality were considered to have less available data, and for simplification and feasibility, do not compose our proposed analytical tool. In public policies and urban health research, due to the historical exclusion of minorities and low priority of social justice concerns, these are characteristics that are still understudied, especially in quantitative studies, which explains the lack of data. Unfortunately, as the proposed tool shall be based on available data, Diversity, and Vitality will not compose its structure. Nevertheless, it is indicated as a major concern in session "3.3 A structured tool for gender-sensitive urban health research and policymaking in Latin America" that these characteristics should be of concern in all policymaking and research

The three Dimensions were defined originally as (free translation from the original): (1) **Proximity** refers to the quality of the space that captures whether people can meet and relate to each other, access facilities and services, and the easiness of use of public transport and shops. It considers that all daily activities should be reachable by walking and public transportation, considering the reasonable distance and time allocation for all types of people, guaranteeing different uses of space; (2) **Autonomy** captures how places provide people with feelings and perception of safety, without restrictions to their use by anyone, regardless of one's physical characteristics; and (3) **Representativeness** assesses visibility, in both material and symbolic ways, highlighting the community's memory and social and cultural heritage and fostering community participation in urban policy decisions[39–41].

Table 1. Publications included in the Literature Review

Full reference	Type	Year	Country	Study design	Language	Main subject
[11]Madariaga, I.S. de Urbanismo Con Perspectiva de Género; Istituto A.; Escandón Impresores: Madrid, 2004; ISBN 847921-101-6.	Handbook	2004	Spain	Theoretical review and case-study	Spanish	Guidebook to help incorporate gender into urban planning, policymaking including especially housing and safety
[25]Sánchez, P.G.; Martínez, M.I. Género y Habitat: Herramientas Para La Acción; Trujillo, C.H., Loustau, A.C., Samos, A.E., Eds.; UN-HABITAT/ROLAC: Rio de Janeiro, 2005; ISBN 92-1-331060-9.	Handbook	2005	N/a	Theoretical review and secondary data analysis	Spanish	International guidelines on gender approaches to the management of urban projects, including a glossary of terms, definitions and concepts
[30]Fenster, T. The Right to the Gendered City: Different Formations of Belonging in Everyday Life. *J. Gend. Stud.* **2005**, *14*, 217–231, doi:10.1080/09589230500264109.	Journal Article	2005	N/a	Theoretical review	English	Theoretical review on women's daily life in relation to citizenship and the right to the city
[9]Rainero, Liliana; Rodigou, Maite; Perez, S. Herramientas Para La Promoción de Ciudades Seguras Desde La Perspectiva Del Género; CISCSA: Cordoba, 2006; ISBN 9789879687826	Handbook	2006	Multiple Latin American countries	Theoretical review, secondary data analysis and case study	Spanish	Theoretical review on urban safety and violence towards women in Latin America and guidelines for urban interventions
[31]Peake, L. Urban Geography: Gender in the City. In The International Encyclopaedia of Human Geography; Elsevier: Toronto, 2009; pp. 320–327.	Book chapter	2009	N/a	Literature review	English	Examines what foci gender research adopts, according to western cities and Global South approaches in urban planning and design
[7]Day, K. Feminist Approaches to Urban Design. In Companion to Urban Design; Banerjee, T., Loukaitou-Sideris, A., Eds.; Routledge, 2011; pp. 150–161.	Book chapter	2011	N/a	Theoretical review	English	Theoretical review on women´s use of public spaces, especially regarding women safety and in transportation
[26]Chestnutt, R.; Ganssauge, K.; Willecke, B.; Baranek, E.; Bock, S.; Huning, S.; Schröder, A.; Damrat, A.; Dorsch, P.; Hofmann, S.; et al. Gender Mainstreaming in Urban Development: Berlin Handbook. 2011	Handbook	2011	Germany	Theoretical review and case-study	English	Gender Mainstreaming and Gender Planning from urban development to mobility, criteria and guidelines for decision- making in gender-sensitive planning at various levels
[27]Muxí, Z.M.; Casanovas, R.; Ciocoletto, A.; Fonseca, M.; Valdivia, B.G. ¿Qué Aporta La Perspectiva de Género Al Urbanismo? Feminismo/s 2011, 17, 105–129.	Journal Article	2011	Spain	Theoretical review and policy analysis	Spanish	Theoretical review on the feminist contributions to urbanism and analysis of Spain related legal framework

Table 1. Publications included in the Literature Review (cont.)

Full reference	Type	Year	Country	Study design	Language	Main subject
[12]Madariaga, I.S. de Mobility of Care: Introducing New Concepts in Urban Transport. In Fair Shared Cities: The Impact of Gender Planning in Europe; Routledge, 2013; pp. 49–69.	Book chapter	2013	N/a	Theoretical review	English	Introduces the concept of Mobility of Care based on the notions of care work and of gender divisions of labour
[39]Ciocoletto, A. Espacios Para La Vida Cotidiana. Auditoría de Calidad Urbana Con Perspectiva de Género. Editor. Comanegra 2014, 73.	Handbook	2014	Spain	Protocol for participatory urban space auditing - primary data collection method	Spanish	Structured tool for participatory urban space auditing with a feminist urbanism perspective
[40]Ciocoletto, A. Urbanismo para la Vida Cotidiana: Herramientas de Análisis y Evaluación Urbana a Tesis Doctoral, Universidad Politécnica de Cataluña, 2014.	Thesis	2014	Spain	PhD thesis on the development of a protocol for participatory urban space auditing	Spanish	Structured tool for participatory urban space auditing with a feminist urbanism perspective
[41]Ciocoletto, A.; Col-lectiu Punt 6 Guía de Reconocimiento Urbano Con Perspectiva de Género. 2014, 1–65.	Handbook	2014	Spain	Protocol for participatory urban space auditing - primary data collection method	Spanish	Structured tool for participatory urban space auditing with a feminist urbanism perspective
[10]Gong, Y.; Palmer, S.; Gallacher, J.; Marsden, T.; Fone, D. A Systematic Review of the Relationship between Objective Measurements of the Urban Environment and Psychological Distress. Environ. Int. 2016, 96, 48–57, doi:10.1016/j.envint.2016.08.019.	Journal Article	2016	N/a	Literature review	English	Literature review on urban environment effects to population mental health
[33]Soto-Villagrán, P. Repensar El Hábitat Urbano Desde Una Perspectiva de Género. Debates, Agendas y Desafíos. *Andamios* **2016**, *13*, 37–56, doi:10.29092/uacm.v13i32.524.	Journal Article	2016	N/a	Theoretical review	Spanish	Theoretical review on feminist approaches to urban environments, and contributions to research on the urban habitat and the gender category in Latin America
[6]Perla Gómez Gallardo Ciudades Seguras Para Mujeres y Niñas. Dfensor - Rev. Mens. la Com. Derechos Humanos del Dist. Fed. 2017, 1, 68.	Full journal issue	2017	Mexico	Secondary data analysis	Spanish	Right to the city, safe cities for women and girls, feminist urbanism

Table 1. Publications included in the Literature Review (cont.)

Full reference	Type	Year	Country	Study design	Language	Main subject
[13]Scheiner, J.; Holz-Rau, C. Women's Complex Daily Lives: A Gendered Look at Trip Chaining and Activity Pattern Entropy in Germany. Transportation (Amst). 2017, 44, 117–138, doi:10.1007/s11116-015-9627-9.	Journal Article	2017	Germany	Theoretical review and secondary data analysis	English	Analysis of women's complex activity patterns compared to men's and respective travel behaviour
[32]Falú, A.M.; Echavarri, L.; Tello Sánchez, F.; García Pizarro, M.; Valle García, J. Guía Para La Planificación Estratégica Local Con Enfoque de Género; 1 ed.; Proyecto UIM y AECID: Córdoba, 2017; ISBN 9789874210548.	Book	2017	Multiple Latin American countries	Theoretical review and primary data collection and analysis based on participatory strategies	Spanish	Presents practices and experiences established in local territories from the GENÉRALO Program, providing a guide for government-society interactions for gender-sensitive strategic urban-planning
[34]Horelli, L. Engendering Urban Planning in Different Contexts–Successes, Constraints and Consequences. Eur. Plan. Stud. 2017, 25, 1779–1796, doi:10.1080/09654313.2017.1339781.	Journal Article	2017	Multiple European countries	Theoretical review and case-studies	English	Analyzes the results of a framework for engendering urban planning with tested examples
[37]Soto Villagrán, P. Hacia La Construcción de Unas Geografías de Género de La Ciudad: Formas Plurales de Habital y Significar Los Espacios Urbanos En Latinoamérica. Rev. Perspect. Geográfica 2018, 23, 13–31.	Journal Article	2018	N/a	Literature review	Spanish	Review of the main contributions of gender and feminist perspective in urban theory and research in Latin America, organizing theoretical concepts, thematic nuclei and research problems
[28]Riaño, B.P.; Rivas, M.R. Ciudades Igualitárias: Guía Práctica de Urbanismo y Género; 2nd ed.; Área de Gobierno de Desarollo Urbano Sostenible del Ayuntamiento de Madrid: Madrid, 2019; ISBN 978-84-7812-778-8.	Book	2019	Spain	Theoretical review and guidelines	Spanish	Handbook to help incorporate gender into urban planning, policymaking in a broad equality and feminist urbanism perspective
[8]Rodó-De-Zárate, M.; I Castany, J.E.; Eizagirre, N. Configuration and Consequences of Fear in Public Space from a Gender Perspective. *Rev. Esp. Investig. Sociol.* **2019**, *167*, 89–105, doi:10.5477/cis/reis.167.89.	Journal Article	2019	Spain	Literature review and qualitative work (Participatory Action Research and Relief Maps)	Spanish	The configuration of fear perception and what kind of implications it has for women

Table 1. Publications included in the Literature Review (cont.)

Full reference	Type	Year	Country	Study design	Language	Main subject
[21]Power, E.R.; Mee, K.J. Housing: An Infrastructure of Care. *https://doi.org/10.1080/02673037.2019.1612038* **2019**, *35*, 484–505, doi:10.1080/02673037.2019.1612038.	Journal Article	2019	N/a	Theoretical review	English	Definitions related to housing as an infrastructure of care, through materialities, markets and governance
[14]Faria, G.C. Cidades Possíveis: Espaço e Gênero Em Escolhas de Mobilidade Urbana, Universidade Federal de Minas Gerais, 2019, Vol. 1. .	Dissertation	2019	Brazil	Theoretical review, primary qualitative data collection and analysis and secondary data analysis	Portuguese	Analysis of women's travel choices and behaviour compared to men's and between different women
[22]Pellegrino, L.K. Habitar Con Justicia Espacial y Equidad de Género. ¿Cuál Es La Imagen de Una Ciudad y Una Vivienda Feminista? In Proceedings of the CHI.IEH (Centro Hábitat Inclusivo). Instituto de la Espacialidad Humana (IEH). UBA - FADU; 2019; pp. 2478–2495.	Conference Proceedings	2019	Multiple	Theoretical review and case-studies	Spanish	Traces the background of projects that address the issue of collective housing and "domestic living" from a gender-sensitive perspective
[15]Ravensbergen, L.; Buliung, R.; Laliberté, N. Toward Feminist Geographies of Cycling. Geogr. Compass 2019, 13, 1–24, doi:10.1111/gec3.12461.	Journal Article	2019	N/a	Literature review	English	Reviews the literature about gender and cycling and critically assesses existing approaches to the topic
[20]Tribouillard, C.; Mastellaro, C. Auditoría de Seguridad de Género y "Caminabilidad": El Nuevo Programa de João Pessoa Con Una Mirada de Inclusión Available online: https://blogs.iadb.org/ciudades-sostenibles/es/auditoria-de-seguridad-de-genero-y-caminabilidad-el-nuevo-programa-de-joao-pessoa-con-una-mirada-de-inclusion/ (accessed on 29 June 2023).	Media news/blog	2019	Brazil	Primary data collection	Spanish	Analysis of a gender inclusion program in Urban Development and City Management policymaking, focused on low-income areas
[38]Power, E.R.; Williams, M.J. Cities of Care: A Platform for Urban Geographical Care Research. *Geogr. Compass* **2020**, *14*, 1–12, doi:10.1111/gec3.12474.	Journal Article	2020	N/a	Theoretical review	English	Theoretical review on urban care research
[19]Montoya-Robledo, V.; Montes Calero, L.; Bernal Carvajal, V.; Galarza Molina, D.C.; Pipicano, W.; Peña, A.J.; Pipicano, C.; López Valderrama, J.S.; Fernández, M.A.; Porras, I.; et al. Gender Stereotypes Affecting Active Mobility of Care in Bogotá. *Transp. Res. Part D Transp. Environ.* **2020**, *86*, 102470, doi:10.1016/j.trd.2020.102470.	Journal Article	2020	Colombia	Theoretical review and secondary data analysis	English	Explores how gender stereotypes intersect with the mobility patterns of women and men cyclists who take children to school

Table 1. Publications included in the Literature Review (cont.)

Full reference	Type	Year	Country	Study design	Language	Main subject
[18]Montoya-Robledo, V.; Escovar-Álvarez, G. Domestic Workers' Commutes in Bogotá: Transportation, Gender and Social Exclusion. *Transp. Res. Part A Policy Pract.* **2020**, *139*, 400–411, doi:10.1016/j.tra.2020.07.019.	Journal Article	2020	Colombia	Theoretical review, secondary data analysis, primary data collection (interviews) and analysis	English	Analyzes domestic workers' daily commutes, focusing on the patterns and characteristics of their trips and the financial implications for their restricted family budget
[29]Terraza, H.; Orlando, M.B.; Lakovits, C.; Lopes Janik, V.; Kalashyan, A. Handbook for Gender-Inclusive Urban Planning and Design; The World Bank: Washington D.C., USA, 2020;	Handbook	2020	N/a	Theoretical review and secondary data analysis	English	Presents a theoretical review on the relationships between gender inequality, the built environment, and urban planning and design, setting guidelines on inclusive cities best practices
[24]Gutiérrez, B.V. La Ciudad Cuidadora: Calidad de Vida Urbana Desde Una Perspectiva Feminista, Universitat Politecnica de Catalunya Barcelonatech, 2021.	Thesis	2021	Spain	Theoretical review and case-study	Spanish	Urban quality of life under a feminist urbanism perspective and a case study with build environment assessment tool
[35]Castillo, A.A.; Moreno Sanchez, L.; Riveros Monsalve, N. Women's Embodied Experiences: Qualitative Tools for a Gender-Conscious Approach to the Territory. *Loci Communes* **2021**, *1*, 1–24, doi:10.31261/lc.2021.01.06.	Journal Article	2021	Mexico and Chile	Theoretical review, primary data collection and analysis, methodological proposal	English	Theoretical review and methodological proposal of the Multidimensional Model of Gender-Conscious Urbanism
[36]Peake Linda; Elsa, K.; Tanyildiz Gökbörü Sarp, R.R.N.; Darren, P. A Feminist Urban Theory for Our Time: Rethinking Social Reproduction and the Urban; Wiley & Sons. Series Antispode Books: Hoboken, NJ, 2021; ISBN 978-1119789154.	Book	2021	Multiple	Theoretical review and diverse methodological approches in different chapters	English	Addresses diverse discussions on social reproduction from the perspective of women's stories and their everyday life struggles in urban contexts
[16]Soto-Villagrán, P. Paisajes Del Cuidado En La Ciudad de México. Experiencias, Movilidad e Infraestructuras. *Íconos - Rev. Ciencias Soc.* **2022**, *XXVI*, 57–75, doi:10.17141/iconos.73.2022.5212.	Journal Article	2022	Mexico	Theoretical review, secondary data analysis, primary data collection (mobile ethnographies) and analysis	Spanish	Analyzes the relationships between mobility, care, and gender through the concept of "landscapes of care"
[17]Jirón, P.A.; Solar-Ortega, M.; Rubio, M.D.; Cortés	Journal	2022	Chile	Theoretical	Spanish	Analyzes the inivisble aspects of care,

Susana, Cid, B.; Carrasco, J. La Espacialización de Los Cuidados. Entretejiendo Relaciones de Cuidado a Través de La Movilidad. *INVI* **2022**, *37*, 199–299, doi:10.5354/0718-8358.2022.65647.	Article	review and secondary data analysis	contributing to the understanding of spacialization and collectivization of care

REFERENCES

1. Sukhera J. Narrative Reviews: Flexible, Rigorous, and Practical. *J Grad Med Educ*. 2022;14(4):414-417. doi:10.4300/JGME-D-22-00480.1
2. Ferrari R. Writing narrative style literature reviews. *Med Writ*. 2015;24(4):230-235. doi:10.1179/2047480615z.000000000329
3. Torraco RJ. Writing Integrative Literature Reviews: Guidelines and Examples. *Hum Resour Dev Rev*. 2005;4(3):356-367. doi:10.1177/1534484305278283
4. Sukhera J. Narrative Reviews in Medical Education: Key Steps for Researchers. *J Grad Med Educ*. 2022;14(4):418-419. doi:10.4300/JGME-D-22-00481.1
5. Baethge C, Goldbeck-Wood S, Mertens S. SANRA—a scale for the quality assessment of narrative review articles. *Res Integr Peer Rev*. 2019;4(1):1-8. doi:10.1186/s41073-019-0064-8
6. Perla Gómez Gallardo. Ciudades seguras para mujeres y niñas. *Dfensor - Rev Mens la Com Derechos Humanos del Dist Fed*. 2017;1(Enero):68.
7. Day K. Feminist approaches to urban design. In: Banerjee T, Loukaitou-Sideris A, eds. *Companion to Urban Design*. Routledge; 2011:150-161.
8. Rodó-De-Zárate M, I Castany JE, Eizagirre N. Configuration and consequences of fear in public space from a gender perspective. *Rev Esp Investig Sociol*. 2019;167:89-105. doi:10.5477/cis/reis.167.89
9. Rainero, Liliana; Rodigou, Maite; Perez S. *Herramientas Para La Promoción de Ciudades Seguras Desde La Perspectiva Del Género*. Cordoba: CISCSA; 2006.
10. Gong Y, Palmer S, Gallacher J, Marsden T, Fone D. A systematic review of the relationship between objective measurements of the urban environment and psychological distress. *Environ Int*. 2016;96:48-57. doi:10.1016/j.envint.2016.08.019
11. Madariaga IS de. *Urbanismo Con Perspectiva de Género*. Istituto A. Madrid: Escandón Impresores; 2004.
12. Madariaga IS de. Mobility of Care: Introducing New Concepts in Urban Transport. In: *Fair Shared Cities: The Impact of Gender Planning in Europe*. 1st ed. Routledge; 2013:49-69.
13. Scheiner J, Holz-Rau C. Women's complex daily lives: a gendered look at trip chaining and activity pattern entropy in Germany. *Transportation (Amst)*. 2017;44(1):117-138. doi:10.1007/s11116-015-9627-9

14. Faria GC. Cidades Possíveis: Espaço e Gênero em Escolhas de Mobilidade Urbana. *Univ Fed Minas Gerais*. 2019;1(1).
15. Ravensbergen L, Buliung R, Laliberté N. Toward feminist geographies of cycling. *Geogr Compass*. 2019;13(7):1-24. doi:10.1111/gec3.12461
16. Soto-Villagrán P. Paisajes del cuidado en la Ciudad de México. Experiencias, movilidad e infraestructuras. *Íconos - Rev Ciencias Soc*. 2022;XXVI(73):57-75. doi:10.17141/iconos.73.2022.5212
17. Jirón PA, Solar-Ortega M, Rubio MD, Cortés Susana, Cid B, Carrasco J. La espacialización de los cuidados. Entretejiendo relaciones de cuidado a través de la movilidad. *INVI*. 2022;37(104):199-299. doi:10.5354/0718-8358.2022.65647
18. Montoya-Robledo V, Escovar-Álvarez G. Domestic workers' commutes in Bogotá: Transportation, gender and social exclusion. *Transp Res Part A Policy Pract*. 2020;139(July 2019):400-411. doi:10.1016/j.tra.2020.07.019
19. Montoya-Robledo V, Montes Calero L, Bernal Carvajal V, et al. Gender stereotypes affecting active mobility of care in Bogotá. *Transp Res Part D Transp Environ*. 2020;86(88):102470. doi:10.1016/j.trd.2020.102470
20. Tribouillard C, Mastellaro C. Auditoría de seguridad de género y "caminabilidad": el nuevo programa de João Pessoa con una mirada de inclusión. BID Mejorando Vidas. https://blogs.iadb.org/ciudades-sostenibles/es/auditoria-de-seguridad-de-genero-y-caminabilidad-el-nuevo-programa-de-joao-pessoa-con-una-mirada-de-inclusion/. Published 2019. Accessed June 29, 2023.
21. Power ER, Mee KJ. Housing: an infrastructure of care. *https://doi.org/101080/0267303720191612038*. 2019;35(3):484-505. doi:10.1080/02673037.2019.1612038
22. Pellegrino LK. Habitar con Justicia espacial y equidad de género. ¿Cuál es la imagen de una ciudad y una vivienda feminista? In: *CHI.IEH (Centro Hábitat Inclusivo). Instituto de La Espacialidad Humana (IEH). UBA - FADU.* ; 2019:2478-2495. https://publicacionescientificas.fadu.uba.ar/index.php/actas/article/view/1178/1608. Accessed May 17, 2021.
23. Damyanovic D, Reinwald F, Weikmann A. Gender Mainstreaming in Urban Planning and Urban Development. 2013:99. https://www.wien.gv.at/stadtentwicklung/studien/pdf/b008358.pdf.
24. Gutiérrez BV. La Ciudad Cuidadora: Calidad de vida Urbana desde una

perspectiva feminista. *Programa Dr Gestión y Valoración Urbana y Arquit ETSAB*. 2021.

25. Sánchez PG, Martínez MI. *Género y Habitat: Herramientas Para La Acción*. (Trujillo CH, Loustau AC, Samos AE, eds.). Rio de Janeiro: UN-HABITAT/ROLAC; 2005.
26. Chestnutt R, Ganssauge K, Willecke B, et al. *Gender Mainstreaming in Urban Development*. (Women's Advisory Committee of the Senate Department for Urban Development, ed.). Berlin: Kulturbuch-Verlag GmbH; 2011.
27. Muxí ZM, Casanovas R, Ciocoletto A, Fonseca M, Valdivia BG. ¿Qué aporta la perspectiva de género al urbanismo? *Feminismo/s*. 2011;17:105-129.
28. Riaño BP, Rivas MR. *Ciudades Igualitárias: Guía Práctica de Urbanismo y Género*. 2nd ed. Madrid: Área de Gobierno de Desarollo Urbano Sostenible del Ayuntamiento de Madrid; 2019.
29. Terraza H, Orlando MB, Lakovits C, Lopes Janik V, Kalashyan A. *Handbook for Gender-Inclusive Urban Planning and Design*. Washington D.C., USA: The World Bank; 2020. doi:10.1596/33197
30. Fenster T. The right to the gendered city: Different formations of belonging in everyday life. *J Gend Stud*. 2005;14(3):217-231. doi:10.1080/09589230500264109
31. Peake L. Urban Geography: Gender in the City. In: *The International Encyclopaedia of Human Geography*. Toronto: Elsevier; 2009:320-327. https://www.researchgate.net/publication/288737279.
32. Falú AM, Echavarri L, Tello Sánchez F, García Pizarro M, Valle García J. *Guía Para La Planificación Estratégica Local Con Enfoque de Género*. 1 ed. Córdoba: Proyecto UIM y AECID; 2017.
33. Soto-Villagrán P. Repensar el hábitat urbano desde una perspectiva de género. Debates, agendas y desafíos. *Andamios*. 2016;13(32):37-56. doi:10.29092/uacm.v13i32.524
34. Horelli L. Engendering urban planning in different contexts–successes, constraints and consequences. *Eur Plan Stud*. 2017;25(10):1779-1796. doi:10.1080/09654313.2017.1339781
35. Castillo AA, Moreno Sanchez L, Riveros Monsalve N. Women's Embodied Experiences: Qualitative Tools for a Gender-Conscious Approach to the Territory. *Loci Communes*. 2021;1(1):1-24. doi:10.31261/lc.2021.01.06

36. Peake Linda, Elsa K, Tanyildiz Gökbörü Sarp RRN, Darren P. *A Feminist Urban Theory for Our Time: Rethinking Social Reproduction and the Urban*. Hoboken, NJ: Wiley & Sons. Series Antispode Books; 2021.
37. Soto Villagrán P. Hacia la construcción de unas geografías de género de la ciudad: formas plurales de habital y significar los espacios urbanos en Latinoamérica. *Rev Perspect Geográfica*. 2018;23(2):13-31.
38. Power ER, Williams MJ. Cities of care: A platform for urban geographical care research. *Geogr Compass*. 2020;14(1):1-12. doi:10.1111/gec3.12474
39. Ciocoletto A. Espacios para la vida cotidiana. Auditoría de Calidad Urbana con perspectiva de Género. *Editor Comanegra*. 2014:73.
40. Ciocoletto A. Urbanismo para la Vida Cotidiana: Herramientas de análisis y evaluación urbana a Tesis Doctoral. 2014.
41. Ciocoletto A, Col-lectiu Punt 6. Guía de reconocimiento urbano con perspectiva de género. 2014:1-65. http://www.punt6.org/wp-content/uploads/2016/08/PDF-mujeres-baja-con-portada.pdf.
42. Niederberger M, Spranger J. Delphi Technique in Health Sciences: A Map. *Front Public Heal*. 2020;8(September):1-10. doi:10.3389/fpubh.2020.00457

Supplementary Material 2

Urban transformation interventions' evaluation studies

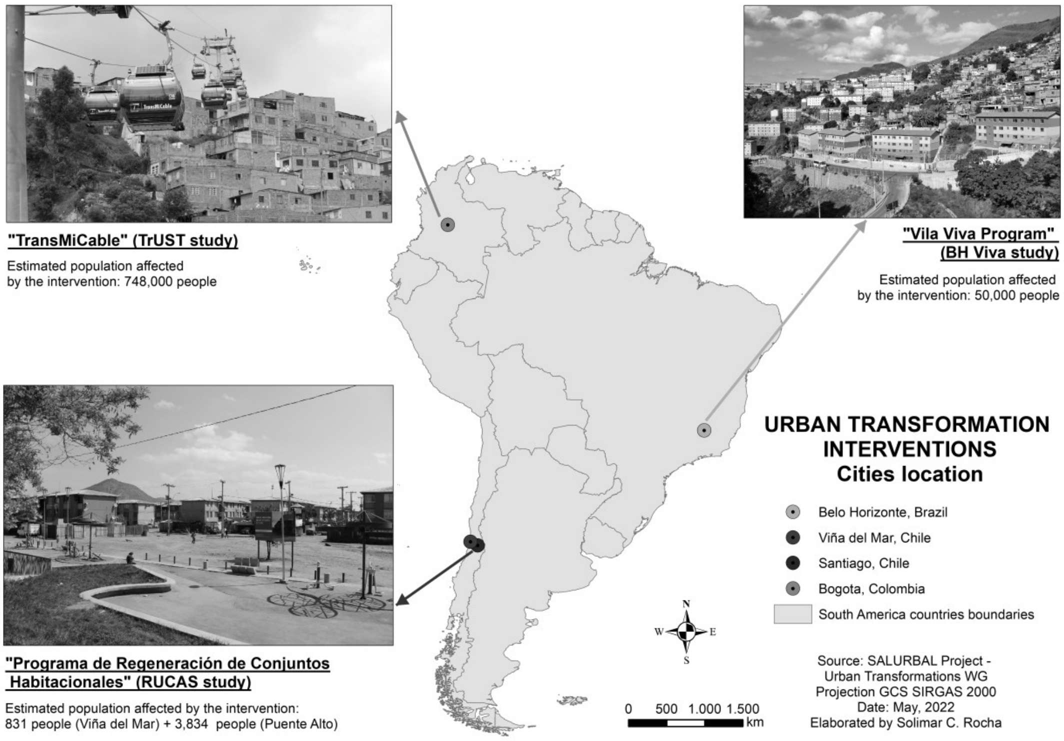

Figure 1. **Urban transformation interventions' locations and respective evaluation studies.**

A. The PAC Programa Vila Viva (PVV) and the BH Viva study

The PVV in the city of Belo Horizonte, Brazil, is the result of a partnership between the Belo Horizonte City Hall and the Federal Government which has been in progress since 2005. The PVV is planned according to the "Plano Global Específico (PGE)" which establishes guidelines to what are the priority interventions for each area. The general priorities summarized by Silveira[1] were related to the sanitary environment, opening, widening, and paving of streets and alleys, improvements in public transportation, health, and education infrastructures, and land tenure regularization. Vila Viva interventions also included improvements in housing and infrastructure, public facilities, and promoted social programs and community involvement [2,3].

The PVV was carried out during a time when a progressive left-wing federal government allocated resources to policies and programs to diminish poverty. In this context, the municipal government of Belo Horizonte seized this opportunity to also allocate budget and effort towards promoting structural interventions. This case presents Aglomerado da Serra, the largest favela in Belo Horizonte and the first territory to undergo PVV interventions[4].

The BH Viva study evaluates PVV health and wellbeing effects in intervention areas with a multimethod approach, drawing on primary and secondary data. For the present analysis, 2017/2018 indicators from three instruments was considered: household surveys, qualitative semi-structured interviews, and Systematic Social Observation [2,5], as presented in Table 1.

B. The Programa de Regeneración de Conjuntos Habitacionales and the Regeneración Urbana, Calidad de Vida y Salud (RUCAS) study

To alleviate the quantitative housing deficit, more than 200,000 housing units were built in Chile in "villas" of three- and four-story blocks of apartments in most of Chile's medium and large cities between 1980 and 2000. Today, more than one million people reside in these areas. However, due to issues with poor construction and rapid deterioration, the focus shifted to addressing the qualitative deficit in housing and neighborhoods. Initially, this involved massive demolitions and the relocation of residents. Subsequently, in 2013, the approach evolved to include the Housing Complex Regeneration Program[6–8].

The program conducted by the Chilean Ministry of Housing and Urbanism (MINVU) is an integral urban redevelopment intervention to improve the quality of life in social housing neighborhoods. The program includes the implementation of green spaces, construction of leisure areas, repaving of streets and sidewalks, installation of bus stops, renovation or enlargement of apartments, and the construction of new housing units in each intervened “villas”[9].

Capitalizing on this intervention, the Regeneración Urbana, Calidad de vida y Salud - RUCAS study aims to evaluate the effects of the intervention on the health and wellbeing of the residents of two “villas” in central Chile. RUCAS uses a longitudinal design to follow two cohorts (one in Viña del Mar and another in Puente Alto) as the intervention unfolds, collecting primary data with repeated measures using different data collection instruments: household surveys, intra-domiciliary observation tool

(IDOT), intra-domiciliary hygrothermal conditions, qualitative methods, and System for Observing Play and Recreation in Communities (SOPARC)[9,10,11]. Here we analyzed indicators from the household survey, the IDOT, the qualitative study, and the SOPARC, as presented in Table 1.

C. The TransMiCable project and the Transformaciones Urbanas y Salud: El caso de TransMiCable (TrUST) study

Ciudad Bolivar is a self-built area located in the southwestern peripheries of Bogota; people living in this area have low access to opportunities, limited access to public services, and transportation options[12]. However, the community has been actively engaged in a prolonged advocacy process to improve their living conditions[13,14]. The installation of the aerial cable car TransMiCable is the result of a participatory and intersectoral integral project, which was inaugurated in 2018 and connects Ciudad Bolívar to the rest of the Bus-Rapid-Transport system of Bogotá. The project was developed as a comprehensive transport intervention that seeks to produce significant urban transformation, implementing enhancements in the area such as the upgrading of public parks, community centers, and recreational facilities[15].

TrUST, is a quasi-experimental study using a mixed-methods approach. The intervention area comprises neighborhoods within an 800-meter airline buffer around the current TransMiCable stations. The control area comprises neighborhoods within an 800-meter airline buffer around the TransMiCable stations projected for future implementation in San Cristobal (an area with socioeconomic and topographic characteristics similar to Ciudad Bolívar) [15]. For the analysis presented in this article, we considered indicators from the household survey, the Our Voice citizen method[16,17], and the System for Observing Play and Recreation in Communities (SOPARC)[11], as presented in Table 1.

Table 1 Samples and data sources of the BH Viva, RUCAS and TrUST studies:

Assessment study	Household survey sample	Qualitative data sample	Systematic Social Observation
**BH Viva study**	**T0 and T1**: 2017<br>N= 401 adults<br>(259 women, 142 men)	**T0 and T1: 2017**<br>N= 45 semi-structured interviews.	**T0 and T1: 2019**<br>Systematic Social Observation during 11 days in 65 streets and 39 alley segments in Aglomerado da Serra (average length 84m, close to interviewees households).
**RUCAS study**	**T0 in Puente Alto: 2019**<br>N= 718 adults<br>(609 women, 109 men)<br><br>**T1 in Viña del Mar: 2018**<br>N= 238 adults<br>(173 women, 65 men)	**T0 in Puente Alto: 2018**<br>N= 2 focus groups (n=19)<br><br>**T1 in Viña del Mar: 2018**<br>N= 8 semi-structured interviews; 2 focus groups (n=12)	**T0 in Puente Alto: 2019**<br>Systematic observation during 7 days (mornings and afternoons) using SOPARC methodology to assess physical activity in 2 parks (total area 13,271 m^2 overall) and 2 multipurpose courts (total area 1.595 m^2).
**TrUST study**<br><br>T0=2018 and T1=2019	**T0: 2018**<br>N= 1031 adults<br>(668 women, 363 men)<br><br>**T1: 2019**<br>N= 825 adults<br>(540 women, 285 men)	**Citizen Science 'Our Voice' Community walks: (2018-2020)**<br>T0: N=11; T1: N=14<br><br>**Community meetings: (2018-2020)**<br>T0: N=3; T1: N=7<br><br>**Community meetings with stakeholders**: **(2018-2020)**<br>T0: N=16; N=14	**T0 and T1: 2018 and 2019**<br>Systematic observations during 7 days (mornings and afternoons) using SOPARC methodology in the Illimaní park, Ciudad Bolívar (total area: 16,000 m^2).

Abbreviations:
SOPARC System for Observing Play and Recreation in Communities (16,17);
T0: measurements conducted during the pre-intervention period; T1: measurements conducted during the post-intervention period.

REFERENCES

1. Silveira DC, Carmo RF, Da Luz ZMP. Planning in four areas of the Vila Viva Program in the city of Belo Horizonte, Brazil: A documentary analysis. *Cienc e Saude Coletiva*. 2019;24(3):1165-1174. doi:10.1590/1413-81232018243.10942017
2. Friche AA de L, Dias MA de S, Reis PB dos, Dias CS, Caiaffa WT. Urban upgrading and its impact on health: a "quasi-experimental" mixed-methods study protocol for the BH-Viva Project. *Cad Saude Publica*. 2015;31(suppl 1):51-64. doi:10.1590/0102-311x00079715
3. de Salles Dias MA, de Lima Friche AA, Mingoti SA, et al. Mortality from homicides in slums in the city of Belo Horizonte, Brazil: An evaluation of the impact of a re-urbanization project. *Int J Environ Res Public Health*. 2019;16(1):1-17. doi:10.3390/ijerph16010154

4. Cardoso AL, Denaldi R. *Urbanização de Favelas No Brasil. Um Balanço Preliminar Do PAC*. Vol 1. 1st ed. (Cardoso AL, Denaldi R, eds.). Rio de Janeiro: Letra Capital; 2018. http://publications.lib.chalmers.se/records/fulltext/245180/245180.pdf %0Ahttps://hdl.handle.net/20.500.12380/245180%0Ahttp://dx.doi.org/10.1016/j.jsames.2011.03.003%0Ahttps://doi.org/10.1016/j.gr.2017.08.001%0Ahttp://dx.doi.org/10.1016/j.precamres.2014.12.
5. Costa DADS, Mingoti SA, Andrade ACDS, Xavier CC, Proietti FA, Caiaffa WT. Indicadores dos atributos físicos e sociais da vizinhança obtidos pelo método de observação social sistemática. *Cad Saude Publica*. 2017;33(8):1-18. doi:10.1590/0102-311X00026316
6. Rodríguez A, Sugranyes A. El problema de vivienda de los “con techo.” *Eure*. 2004;30(91):53-65. doi:10.4067/s0250-71612004009100004
7. MINVU. *Vivienda social en copropiedad*. 1a. ed. Santiago: MINVU; 2014.
8. Ministerio de Vivienda y Urbanismo Chile. Regeneración de Condominios sociales (Recuperación de Condominios Sociales - Segunda Oportunidad). 2018:1-4.
9. Baeza F, Vives Vergara A, González F, et al. The Regeneración Urbana, Calidad de Vida y Salud - RUCAS project: a Chilean multi-methods study to evaluate the impact of urban regeneration on resident health and wellbeing. *BMC Public Health*. 2021;21(1):728. doi:10.1186/s12889-021-10739-3
10. McKenzie TL, Cohen DA, Sehgal A, Williamson S, Golinelli D. System for Observing Play and Recreation in Communities (SOPARC): Reliability and Feasibility Measures. *J Phys Act Heal*. 2006;3(s1):S208-S222. doi:10.1123/jpah.3.s1.s208
11. Santos MPM, Rech CR, Alberico CO, et al. Utility and Reliability of an App for the System for Observing Play and Recreation in Communities (iSOPARC®). *Meas Phys Educ Exerc Sci*. 2016;20(2):93-98. doi:10.1080/1091367X.2015.1120733
12. Guevara-Aladino P, Baldovino-Chiquillo L, Rubio MA, et al. Winds of change: the case of TransMiCable, a community-engaged transport intervention improving equity and health in Bogotá, Colombia. *Cities Heal*. May 2022:1-9. doi:10.1080/23748834.2022.2038981
13. Madrigal A, Bolívar Yudy Sánchez S. Las memorias del conflicto armado y la

violencia en Colombia: Ciudad Bolívar como referente de mantenimiento de memoria colectiva significativa en Bogotá. *Ciudad Paz-ando*. 2012;5(2):71-86. doi:10.14483/2422278X.5356

14. Nicolás A, Rodríguez A, Luis /, et al. Ciudad Bolívar y el cable aéreo: expresiones y trayectorias de la ciudad segregada. *Anekumene*. 2020;(19):35-50. doi:10.17227/Anekumene.2020.num19.13596

15. Sarmiento OL, Higuera-Mendieta D, Wilches-Mogollon MA, et al. Urban Transformations and Health: Methods for TrUST—a Natural Experiment Evaluating the Impacts of a Mass Transit Cable Car in Bogotá, Colombia. *Front Public Heal*. 2020;8:64. doi:10.3389/fpubh.2020.00064

16. King AC, Winter SJ, Chrisinger BW, Hua J, Banchoff AW. Maximizing the promise of citizen science to advance health and prevent disease. *Prev Med (Baltim)*. 2019;119:44-47. doi:10.1016/j.ypmed.2018.12.016

17. King A, Odunitan-Wayas F, Chaudhury M, et al. Community-Based Approaches to Reducing Health Inequities and Fostering Environmental Justice through Global Youth-Engaged Citizen Science. *Int J Environ Res Public Health*. 2021;18(3):892. doi:10.3390/ijerph18030892
